# Supplementary material for: The relationship between auditory processing disorder and articulation disorders
Source: PLoS One. 2025 Oct 10;20(10):e0333016. doi: 10.1371/journal.pone.0333016 (PMC12513641; doi:10.1371/journal.pone.0333016)
Supplement: Attachment — Submitted filename: study-comments-1.docx [file pone.0333016.s003.docx]

| **Reviewer** | **Comments** | **Response** |
| --- | --- | --- |
| 1 | Line 207 (Table 1): The article refers to participants' educational levels (elementary, middle, and high  school) but does not define the corresponding age ranges, which may not be clear for international  readers. This is particularly important because of the unequal distribution between groups, which  could influence the interpretation of the results. | The following paragraph has been added: “The majority of participants were elementary school students (72%). For clarity, educational levels were categorized as follows: elementary (ages 6–10 years), middle school (ages 11–14 years), and high school (ages 15–18 years). Table 1 presents the demographic details of the participants. “ |
| 1 | Line 201 - 206: The reported p-values (p > 0.001) appear inconsistent with the interpretation of  significant differences. For the MLD test, while p < 0.01 is stated, the Z-value (-0.319) suggests minimal differences. Please ensure consistency and clarity in reporting the statistical results. | The values of *p* were rewritten to be 0.05 across the entire paper. Additionally, the statistician misreported the p-values for this section, mistakenly using the smaller and larger symbols incorrectly. These symbols have been corrected. |
| 1 | Line 213: The presentation of information is unclear. The numbers in brackets do not align properlywith the corresponding sound misarticulations. Additionally, the way it is written seems to imply a  Significant difference exist. A more precise and structured presentation is needed to avoid confusionand accurately convey the findings. | The comment has been addressed and the text has been changed to “Based on the classification of sound misarticulations, the number of abnormal cases was higher than normal cases for /r/ (16 cases, 43.2%), /s/ (5 cases, 13.5%), /q/ (3 cases, 8.1%), /k/ (4 cases, 10.8%), /θ/ (4 cases, 10.8%), and /ʤ/ (5 cases, 13.5%). However, no statistically significant association was found between APD and sound misarticulations (*p* = 0.46, Fisher's exact test = 4.58; Table 2). |
| 1 | Example Line 230 and 244 (In results section): It is unclear how it was determined that a child  understood or misunderstood a task. Also, how the CAPD tests were specifically applied to evaluate  misarticulations of individual sounds (Eg., “Out of the ten participants who took the MLD test for the  /s/ sound, 26.5% failed”). Providing a more detailed description of the procedures in the Methods  section would be helpful, or the results could be written more clearly when interpreted. | A paragraph was added: “  Before the APD test, all participants received thorough instructions, repeated up to three times if needed to ensure understanding. Additionally, screening assessments were conducted for each participant before proceeding to the main tests.”  Line (167-172) in the Methods section would further explain the assessment procedure conducted to diagnose the presence of an articulation disorder. |
| 1 | Line 249 and Line 306: The statement attributing temporal information processing to the MLD test and  spectral analysis to the PPS test is inaccurate. The MLD test is primarily a measure of binaural  interaction and PPS is measuring temporal sequencing. While pitch changes in PPS are related to  frequency, the test does not evaluate the listener’s ability to analyze the spectral components of a  sound. I suggest to include relevant citations to justify the connection. | The lines have been revised and altered (Line 243). “However, no statistically significant association was found between abnormal binaural interaction, as measured by the MLD test [27], and sound misarticulations (p = 0.60, Fisher's exact test = 3.95), as depicted in Table 4.”    And (Line 314) “A one-way repeated measures ANOVA with the factor Ear revealed no significant differences in temporal ordering difficulties between both the right and left ear, as measured by the PPS test ^[28]^, attributable to sound misperception (F (1,45) = 0.96; *p*> 0.05; Table 6).”  . |
| 1 | Line 456: What does “struggle” mean in this context? Does it imply that subjects performed poorly or  that they had difficulty understanding the task? | It means they performed poorly. A paragraph has been added (Line 462)to clarify this point: “Additionally, regardless of the type of sound misarticulation, most participants performed worse on the MLD test compared to other components of the auditory processing battery. |
| 1 | Line 461: Poor performance in PPS does not mean that poor discrimination between frequencies. | Thank you for your comment. We have changed the sentence (Line 468) to “Furthermore, our investigation indicated that participants with /r/ and /ʤ/ misarticulations performed poorly on the PPS test, indicating challenges in understanding complex auditory information, pattern recognition, and temporal processing ^[19,28]^” |
| 2 | The manuscript alternates between using "CAPD" and "APD" to refer to central auditory processing  disorder. Consistently using one term throughout the text would improve coherence. | The manuscript has been revised to ensure consistency, with **"APD"** being used instead of **"CAPD"** throughout the text. |
| 2 | The way significance values are reported is inconsistent. Some are written as "p<0.05," while others  are presented as "p value < 0.05." | All the text of significance level has been changed to *P* <0.05 |
| 2 | The phrasing “The statistical significance level was set at α ≤ 0.05” below each Table could be misleading, as it suggests a range rather than a fixed threshold. For clarity, it would be better to state,  α = 0.05. | **The significance level notation under all tables and figures has been revised from α ≤ 0.05 to α = 0.05 for clarity and consistency.** |
| 2 | Line 371: The phrase “Prolong the prognosis” is not commonly used in clinical context and its  meaning is ambiguous. | The text has been changed (Line 379): "The presence of APD in participants with sound misarticulations was found to affect sound perception and extend the duration of the condition." |
| 2 | It does not clarify whether the study participants have been diagnosed with Speech Sound Disorder  (SSD). While the authors mention that the inclusion criterion is misarticulation of specific speech  sounds and refers to articulatory disorder, it remains unclear whether the participants have idiopathic  SSD or SSD resulting from known causes, such as Complex Neurodevelopmental Disorders. Although  the speech manifestations may appear similar, the underlying causes differ significantly. | The sentence (Line 123) was rephrased to “Based on the criteria of ^[1-2]^, participants were diagnosed as having either normal auditory processing or APD. The inclusion criteria were as follows: (a) Normal hearing sensitivity across all tested frequencies; (b) normal middle ear function (type A tympanometry); (c) completion of APD subtests included in Dr. Frank Musiek’s battery; and (d) complaints limited to of misarticulations (pure articulation disorders based on SLP evaluations) of the /r/, /s/, /k/, /θ/, /dʒ/, and /q/ sounds resulting from idiopathic speech sound disorder (SSD). This ensured that the study focused on participants with functional SSD of unknown cause, providing a clear understanding of diagnoses and avoiding confounding from other underlying conditions.” |
| 2 | A key aspect to consider is that the diagnosis of speech sound disorders (SSD) must be thorough  and specify the type of disorder, irrespective of the classification system used (whether Shriberg et al.  or Dodd et al.). It is essential to clarify whether the disorder is cognitive-linguistic (phonological),  involves speech errors (sound distortion), or is related to speech motor. Additionally, SSD is classified  as persistent if it continues beyond the age of 9. Therefore, it is important to clearly distinguish the  analysis of data from children aged 6 years to 18 years. | Thank you for your comment. The disorder in our sample is idiopathic SSD. We acknowledge that SSD is classified as persistent if it continues beyond the age of 9. However, including children aged 6 years in our sample was important for understanding the early development and progression of SSD. By analyzing data from children aged 6 to 18 years, we identified whether central auditory processing skills contribute to the persistence of SSD and developed more effective early intervention strategies. While we did not specifically study the differences between the ages of 6 to 18 years, our approach aimed to provide a broad understanding of SSD across this age range. |
| 2 | When it comes to diagnosing Auditory Processing Disorder (APD), the specific literature  recommends conducting tests for APD starting at the age of 7. It is important to specify the normality  criteria for different age groups for each test, particularly for 6-year-olds. This information should be  clearly explained, and the adopted normality criteria should be presented. | Thank you for your comment. The normative data for each test in the APD battery varies. We have provided the normative data for each test separately in the 'The Battery for APD' subsection. |
| 2 | Page 15: Why do not the authors cite the 2007 paper, which is accessible to all readers? | The study is already cited. You can find it on Line 382 “When the auditory system fails to accurately discriminate and process acoustic characteristics, it may lead to sound distortions in children ^[29]^. At very rapid speech rates, auditory discrimination deteriorates further ^[30]^. As a result, the child may be able to process certain acoustic features of sounds correctly, affecting sound production ^[30]^. Since the auditory cortex is considered tonotopic ^[31]^, it is suspected that speech sound frequencies in participants with abnormal auditory processing may not be sufficiently stimulated within the auditory cortex.” |
| 2 | Page 26-27, lines 388-394: Revise the reference, as the study does not mention hyperactivity. | The paragraph has been revised and modified according to the original study (Line 395)  “The study found that children with SSD, including articulation difficulties, performed poorly on auditory processing tasks. These findings align with the present study, highlighting a potential link between articulation deficits and auditory processing skills. Moreover, the severity of the articulation disorder may influence the extent of auditory processing difficulties, suggesting that more pronounced speech production issues could be associated with greater deficits in auditory discrimination, temporal processing, and auditory memory. However, it is important to distinguish between APD and articulation disorders: APD involves deficits in the brain’s interpretation of auditory information, while articulation disorders are characterized by difficulties in the physical production of speech sounds. Although these conditions may coexist, they represent distinct diagnostic entities requiring separate evaluation and intervention approaches ^[18]^.” |
| 2 | In general, it is important to clarify the results. Were the analyses conducted with participants of all  ages grouped together? Do 6-year-olds perform as well as 18-year-olds in auditory processing tests?  At this developmental stage, does age play a role? Additionally, why were children with only one  speech error not analyzed separately from those with two or more errors? Is there a correlation  between the severity of errors and auditory processing disorders (APD)? Another relevant point  regarding speech sounds is that when the error is phonological, it may occur as a substitution for a  phonological feature or rule of the language. Therefore, it would be interesting to know if everyone  who made errors in the /s/ sound made the same type of error. | A paragraph has been added explaining and interpreting the results (Line 363): “Participants' variables, such as age, gender, and educational level, as shown in Table 1, were considered when conducting the APD test battery. Despite the fact that the auditory system maturation varies with age, the analysis focused on linking specific sound misarticulations with APD. All participants who completed the APD test battery were evaluated using the same passing criteria, regardless of the number or severity of speech sound errors or age differences, provided they were within the target age range for the tests.”  Regarding the presence of a phonological disorder, all participants were confirmed to have only articulation disorders.  Additionally, the type of speech sound errors and the correlation between the severity of errors and APD are beyond the scope of this study, as the participants had misarticulations in one sound and did not have phonological disorders. |
| 2 | Page 16 lines 196-200. It was not specified how many participants exhibited each type of sounderror, which is crucial for understanding the results and supporting the discussion. Additionally, it is  important to identify the type of error observed—whether it was a distortion or a phonological error. Ifit is phonological, we should note the specific phonological process involved. Furthermore, clarifying  whether the errors involved the omission or substitution of sounds would be beneficial. It would alsobe helpful to know how many sounds each participant struggles with, as this information can aid indetermining the severity of the Speech Sound Disorder (SSD). You might consider using the Percent  Consonants Correct (PCC) or the PCC-R, as described by Shriberg et al., to correlate severity withAuditory Processing Disorder (APD). | A paragraph has been added (Line 196): “Participants exhibited two primary types of articulation errors: substitution (n = 28) and distortion (n = 23). Among those with substitution errors, 20 participants (71.4%) had APD, while 8 (28.6%) demonstrated normal auditory processing. Similarly, among participants with distortion errors, 17 (73.9%) had APD, and 6 (26.1%) exhibited normal auditory processing. Overall, 37 of 51 participants (72.5%) were diagnosed with APD, regardless of the type of articulation error, highlighting a strong association between articulation difficulties and auditory processing deficits. Notably, all participants exhibited articulation disorders rather than phonological disorders, as their errors were limited to distortion and substitution rather than phonological rule-based processes.  The Percent Consonants Correct–Revised (PCC-R) metric was used to assess the severity of speech sound errors. PCC-R scores among participants ranged from 76% to 97%, indicating varying degrees of mild to moderate SSD severity. According to Shriberg et al., PCC-R values above 85% indicate a mild disorder, while scores below this threshold suggest moderate impairment ^[26]^.  The PCC-R scores varied depending on the specific sound, with /r/ at 76%, /s/ at 87%, /θ/ at 97%, /q/ at 87%, /k/ at 91%, and /ʤ/ at 90%. This variation reflects differences in production frequency and accuracy for each sound within the sample. Additionally, the severity of errors fluctuated based on the recurrence of each sound in the dataset.  Notably, /r/ is used more frequently in Arabic speech compared to /θ/, which may contribute to the observed differences in PCC-R values. Frequent exposure to /r/ may enhance articulation accuracy, whereas less commonly used phonemes like /θ/ might exhibit higher PCC-R scores due to their lower occurrence and reduced likelihood of errors. |
| 2 | Page 27 lines 395-396 This refers to another communication disorder with distinct characteristics  and symptoms. | The paragraph (Line 295) has been rewritten to be “The study found that children with SSD, including articulation difficulties, performed poorly on auditory processing tasks. These findings align with the present study, highlighting a potential link between articulation deficits and auditory processing skills. Moreover, the severity of the articulation disorder may influence the extent of auditory processing difficulties, suggesting that more pronounced speech production issues could be associated with greater deficits in auditory discrimination, temporal processing, and auditory memory. However, it is important to distinguish between APD and articulation disorders: APD involves deficits in the brain’s interpretation of auditory information, while articulation disorders are characterized by difficulties in the physical production of speech sounds. Although these conditions may coexist, they represent distinct diagnostic entities requiring separate evaluation and intervention approaches ^[18]^.”  . |

****Thank you for your valuable feedback. Please note that the manuscript has been carefully revised to enhance clarity, ensure consistency, and improve the overall flow of the text. We hope these improvements meet your expectations and contribute to a smoother reading experience.**
