# Supplementary material for: The relationship between auditory processing disorder and articulation disorders
Source: PLoS One. 2025 Oct 10;20(10):e0333016. doi: 10.1371/journal.pone.0333016 (PMC12513641; doi:10.1371/journal.pone.0333016)
Supplement: Attachment — Submitted filename: reply to reviewers final minor version.docx [file pone.0333016.s004.docx]

| **Comment Number** | **Original comments of the reviewer** | **Reply by the author(s)** |
| --- | --- | --- |
| Reviewer 1 Comment 1 | Reviewer #1: Thank you for the thoughtful revisions addressing the prior comments. The manuscript is improved overall. However, I still recommend minor revision as stated below.  Abstract (results): The statement “Central auditory processing evaluations revealed abnormalities in 36 of 51 participants (36%)” appears inconsistent with the findings reported in the results section. Please correct or clarify. | Thank you for your comment. The sentence has been revised for accuracy and now reads:  “The central auditory processing evaluations revealed abnormalities in 37 out of 51 participants (72.55%).”This correction aligns with the data presented in the results section.. |
| Reviewer 1 Comment 2 | Line 127: Typo. “complaints limited to of misarticulations (pure articulation disorders based on SLP evaluations) of the /r/..” | Thank you for pointing that out. The sentence on line 127 has been corrected to remove the typo and now reads:  “complaints limited to misarticulations (pure articulation disorders based on SLP evaluations) of the… ” |
| Reviewer 1 Comment 3 | Line 127: “administrated” is incorrect, should be “administered”. | Thank you for your comment. We have reviewed it. |
| Reviewer 1 Comment 4 | Line 260: Please include “ …"binaural" temporal information processing as measured by MLD” to be more accurate. | Thank you for your suggestion. The sentence on line 260 has been revised to include the recommended clarification and now reads:  “…binaural temporal information processing as measured by MLD” to enhance accuracy and specificity. |
| Reviewer 1 Comment 5 | Line 384: Sentence is inconsistent, “ As a result, the child may be able to process certain acoustic features of sounds correctly, affecting sound production.” Please revise for clarity. | Thank you for your comment. We have reviewed the clean version of the manuscript and confirm that the sentence already appears as:  “As a result, the child may struggle to accurately perceive certain acoustic features of sounds, which can negatively affect their ability to produce those sounds correctly.” |
| Reviewer 2 Comment 1 |  | Thank you for your thoughtful feedback. We appreciate your concern regarding the classification of substitution errors. In our study, the substitution errors were limited to a single sound per child and did not follow a broader phonological pattern. Based on this, we classified them as articulatory in nature rather than phonological.  This distinction is supported by Vuolo (2023), who states:  “Children with isolated sound errors that do not follow a rule-based pattern may be more appropriately classified as having articulatory rather than phonological deficits, particularly when the error is consistent and motor-based.”  (Vuolo, J. Articulatory and segmental performance in children with and without speech disorder: A multiple case pilot study. Clinical Linguistics & Phonetics, 2023; 37(10): 935–957. https://doi.org/10.1080/02699206.2022.2108724)  Additionally, the National Center for Biotechnology Information (NCBI) defines phonological disorder as:  “A disorder characterized by the failure to use developmentally expected speech sounds that are appropriate for the individual's age (i.e., the individual makes errors in sound production or use or omits sounds such as final consonants).”  This typically involves systematic, rule-based errors that affect multiple phonemes.  Given that the substitutions in our sample were isolated and not part of a phonological process, we believe the classification as articulatory is appropriate. We have clarified this point in the manuscript to ensure accurate representation of the studied population. A paragraph has been added to the inclusion criteria explaining this point: “These errors were either distortions or single-sound substitutions that did not follow a rule-based phonological pattern, supporting their classification as articulatory in nature. This selection ensured a homogenous sample of children with functional SSD, allowing for a focused investigation of auditory processing abilities without interference from broader phonological or neurological conditions. |
| Reviewer 2 Comment 2 | The PCC-R was then calculated for each sound, which is unusual because it is a metric used for a speech sample where all the sounds of the language can occur. It should be noted that the PCC-R does not treat distortions as errors and therefore mainly indicates phonological errors. For example, the PCC-R value of 97% for the sound /ɵ/ shows that it has no phonological errors, as it is close to the 100% maximum, but we do not know the percentage of errors caused by distortion. | Thank you for the feedback. The PCC-R in this analysis was calculated based solely on substitution errors, in accordance with its standard application. Distortion errors were excluded, which contributed to higher PCC-R values compared to previous calculations where all error types were considered. According to the literature (Shriberg & Kwiatkowski, 1982; Lewis, 1997), distortion is classified as a minimal misarticulation error and is not treated as a phonemic error within the PCC-R framework. Therefore, cases with distortion are considered to have 100% PCC-R, which explains the increase in PCC-R scores observed in this analysis. The updated text is: "The PCC-R and distortion rates varied depending on the specific sound, with /r/ at 86.5% and 24%, /s/ at 93.5% and 13%, /θ/ at 99.25% and 3%, /q/ at 93.5% and 13%, /k/ at 97.0% and 9%, and /ʤ/ at 98.33% and 10%. This variation reflects differences in production frequency and accuracy for each sound within the sample. Additionally, the severity of errors fluctuated based on the recurrence of each sound in the dataset."  References  Shriberg, L. D., & Kwiatkowski, J. (1982). Phonological disorders III: A procedure for assessing severity of involvement. Journal of Speech and Hearing Disorders, 47(3), 256–270. https://doi.org/10.1044/jshd.4703.256  Lewis, B. A. (1997). The percentage of consonants correct (PCC) metric: Extensions and reliability data. Journal of Speech, Language, and Hearing Research, 40(4), 708–722. https://doi.org/10.1044/JSLHR.4004.708  Jartun, R. (1992). The percentage consonants correct and intelligibility of normal, language delayed, and history of language delayed children (Master’s thesis, Portland State University). https://doi.org/10.15760/etd.6212 |
| Reviewer 2 Comment 3 | Clarifying that the children in the study had idiopathic SSD is sufficient, but distinguishing substitution errors from distortions would make the analyses more robust. The results presented are described in greater detail, as is the discussion. The main contribution of this paper is to highlight that, depending on the error—whether phonological or articulatory—a detailed analysis of auditory processing based on the type of error observed in the SSD can support treatment with the | Thank you for your thoughtful feedback. We appreciate your concern regarding the classification of substitution errors and agree that distinguishing them from distortions enhances the robustness of the analysis.  In our study, substitution errors were limited to a single sound per child and did not follow a broader phonological pattern. Based on this observation, we classified these errors as articulatory in nature rather than phonological. This distinction is supported by Vuolo (2023), who states:  “Children with isolated sound errors that do not follow a rule-based pattern may be more appropriately classified as having articulatory rather than phonological deficits, particularly when the error is consistent and motor-based.”  (Vuolo, J. Articulatory and segmental performance in children with and without speech disorder: A multiple case pilot study. Clinical Linguistics & Phonetics, 2023; 37(10): 935–957. https://doi.org/10.1080/02699206.2022.2108724)  Additionally, the National Center for Biotechnology Information (NCBI) defines phonological disorder as:  “A disorder characterized by the failure to use developmentally expected speech sounds that are appropriate for the individual's age (i.e., the individual makes errors in sound production or use or omits sounds such as final consonants).”  This typically involves systematic, rule-based errors that affect multiple phonemes. Given that the substitutions in our sample were isolated and not part of a phonological process, we believe the classification as articulatory is appropriate.  To ensure clarity, we have added a paragraph to the inclusion criteria in the manuscript:  “These errors were either distortions or single-sound substitutions that did not follow a rule-based phonological pattern, supporting their classification as articulatory in nature. This selection ensured a homogenous sample of children with functional SSD, allowing for a focused investigation of auditory processing abilities without interference from broader phonological or neurological conditions.”  We hope this clarification strengthens the rationale behind our classification and supports the integrity of the analysis. |
